# A joint model for lesion segmentation and classification of MS and NMOSD

**DOI:** 10.3389/fnins.2024.1351387

**Published:** 2024-05-27

**Authors:** Lan Huang, Yangguang Shao, Hui Yang, Chunjie Guo, Yan Wang, Ziqi Zhao, Yingchun Gong

**Affiliations:** ^1^College of Computer Science and Technology, Jilin University, Changchun, China; ^2^Public Computer Education and Research Center, Jilin University, Changchun, China; ^3^Department of Radiology, The First Hospital of Jilin University, Changchun, China

**Keywords:** MS, NMOSD, joint model, MRI, disease classification, lesion segmentation

## Abstract

**Introduction:**

Multiple sclerosis (MS) and neuromyelitis optic spectrum disorder (NMOSD) are mimic autoimmune diseases of the central nervous system with a very high disability rate. Their clinical symptoms and imaging findings are similar, making it difficult to diagnose and differentiate. Existing research typically employs the T2-weighted fluid-attenuated inversion recovery (T2-FLAIR) MRI imaging technique to focus on a single task in MS and NMOSD lesion segmentation or disease classification, while ignoring the collaboration between the tasks.

**Methods:**

To make full use of the correlation between lesion segmentation and disease classification tasks of MS and NMOSD, so as to improve the accuracy and speed of the recognition and diagnosis of MS and NMOSD, a joint model is proposed in this study. The joint model primarily comprises three components: an information-sharing subnetwork, a lesion segmentation subnetwork, and a disease classification subnetwork. Among them, the information-sharing subnetwork adopts a dualbranch structure composed of a convolution module and a Swin Transformer module to extract local and global features, respectively. These features are then input into the lesion segmentation subnetwork and disease classification subnetwork to obtain results for both tasks simultaneously. In addition, to further enhance the mutual guidance between the tasks, this study proposes two information interaction methods: a lesion guidance module and a crosstask loss function. Furthermore, the lesion location maps provide interpretability for the diagnosis process of the deep learning model.

**Results:**

The joint model achieved a Dice similarity coefficient (DSC) of 74.87% on the lesion segmentation task and accuracy (ACC) of 92.36% on the disease classification task, demonstrating its superior performance. By setting up ablation experiments, the effectiveness of information sharing and interaction between tasks is verified.

**Discussion:**

The results show that the joint model can effectively improve the performance of the two tasks.

## 1 Introduction

The demyelinating disease of the central nervous system is an autoimmune disease characterized by multifocal and inflammatory demyelination of the central nervous system. Both Multiple sclerosis (MS) and Neuromyelitis optic spectrum disorder (NMOSD) are demyelinating diseases of the central nervous system (Bruscolini et al., 2018; McGinley et al., 2021). MS and NMOSD may be easily confused clinically due to their overlapping features (Yokote and Mizusawa, 2016).

Magnetic Resonance Imaging (MRI) is a commonly used medical imaging technology in clinical practice (Bauer et al., 2013) for prognosis and treatment response evaluation of MS and NMOSD (Filippi et al., 2016; Rotstein and Montalban, 2019). Furthermore, the T2-weighted fluid-attenuated inversion recovery (T2-FLAIR) sequence can inhibit a certain range of fluid signals, thereby reducing the cerebrospinal fluid signal intensity and enhancing the visibility of small brain lesions and periventricular lesions. Therefore, T2-FLAIR sequence imaging plays a crucial role in the diagnosis of cerebral nervous system diseases (Wattjes et al., 2021).

In order to accurately diagnose MS and NMOSD clinically, it is usually necessary for radiologists to manually segment the white matter high signal presented on MRI images, and then diagnose MS according to the McDonald diagnostic criteria (Wattjes et al., 2021) and NMO diagnostic criteria (Griggs et al., 2016) according to the distribution and morphology of lesions and the clinical manifestations of patients. However, the entire diagnostic process is a time-consuming and onerous task for doctors.

Deep learning has achieved advanced performance in image processing due to a large amount of labeled data, enabling accurate diagnosis of MS and NMOSD (Lee et al., 2017; Lundervold and Lundervold, 2019). The MS and NMOSD auxiliary diagnosis based on deep learning mainly includes two tasks: lesion segmentation and disease classification. The task of lesion segmentation involves identifying and segmenting the lesions according to the high white matter signal observed by the patient’s MRI, judging the severity of the patient, and monitoring the course of the disease through quantitative measurement. The task of disease classification aims to accurately diagnose patients, distinguishing between MS and NMOSD according to the shape and distribution characteristics of the lesions.

**Figure 1:**
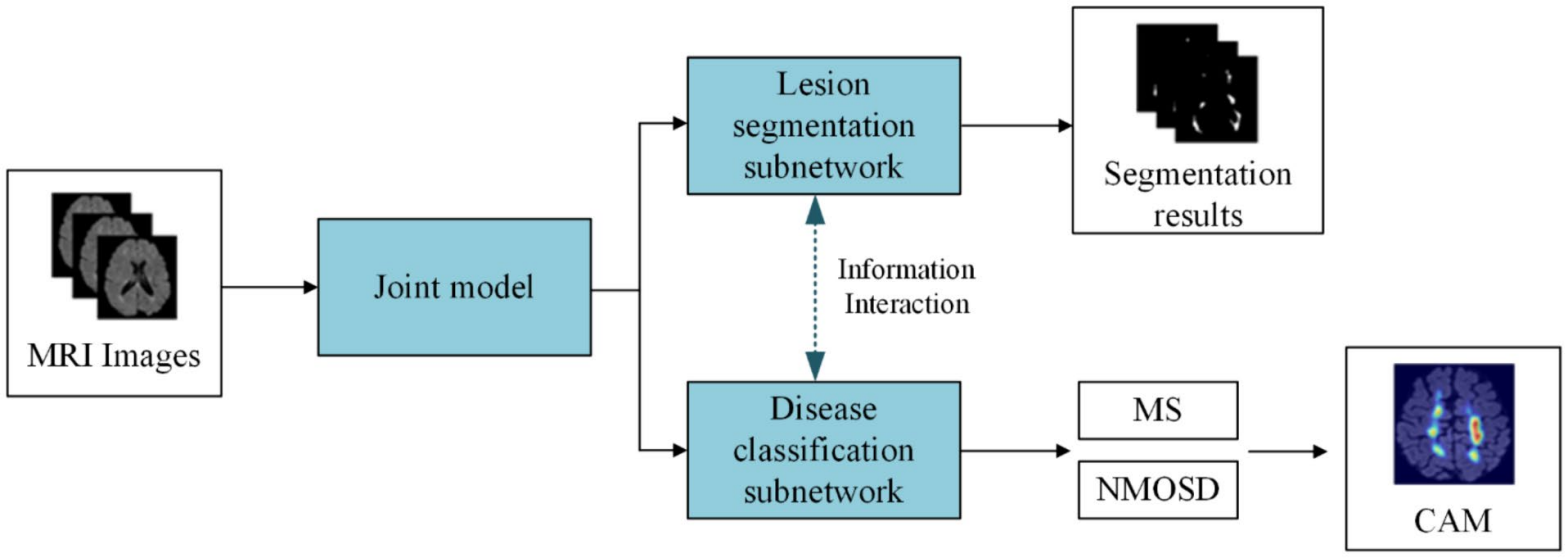
The main work content of this study.

In most research, the segmentation and classification of MS and NMOSD are studied independently. In order to improve the efficiency and accuracy of the auxiliary diagnosis model, this study analyzes the correlation between lesion segmentation and disease classification tasks and combines the existing deep learning technology to carry out the following research work:

(1) We proposed a joint model of lesion segmentation and disease classification of MS and NMOSD, which is based on the intrinsic correlation between the two tasks and used to segment and classify MS and NMOSD simultaneously. The structure of the joint model (Figure 1) mainly includes three components: an information-sharing subnetwork, a lesion segmentation subnetwork, and a disease classification subnetwork.
(2) We proposed two information interaction methods to improve the performance of lesion segmentation and disease classification tasks in a mutually guided manner, one is a lesion guidance module and the other is a cross-task loss function. Moreover, the lesion location maps provide interpretability for the diagnosis of MS and NMOSD.

The subsequent work consists of four sections, each briefly described as follows: in Section 2 we provide an overview of the relevant research on lesion segmentation and disease classification in MS and NMOSD. In Section 3 we offer a detailed presentation of the joint model for MS and NMOSD in this study. In Section 4 we involve experimental validation of the performance of the joint models proposed in this study. In Section 5 we present the conclusions drawn from this study and provide prospects.

## 2 Related works

Deep learning-based auxiliary diagnosis of MS and NMOSD mainly includes two tasks: lesion segmentation and disease classification. The lesion segmentation task involves identifying and segmenting lesions based on high signal white matter in a patient’s MRI, enabling quantitative measurement to assess the severity of the patient’s condition and monitor disease progression. The disease classification task aims to diagnose the specific condition a patient has, distinguishing whether the patient has MS or NMOSD based on the morphology and distribution characteristics of the lesions.

Traditional segmentation methods include threshold segmentation algorithms, region-growing algorithms, edge detection algorithms, and watershed algorithms. Among these, threshold segmentation algorithms enhance images based on the differences in signal intensity between healthy brain tissue and lesion regions. These methods involve setting one or more thresholds manually or using algorithms after preprocessing the images to segment them into different parts based on intensity values. For instance, Wicks et al. (1992) proposed an intensity-based global threshold segmentation method for MS lesion segmentation. Hence, Wang et al. (1998) addressed the impact of scanner sensitivity on thresholds by proposing histogram matching algorithms. Their study demonstrated that histogram matching significantly reduces dependence on threshold selection for lesion segmentation.

In recent years, deep learning methods have exhibited superior performance in the field of image segmentation (Wang et al., 2022). Particularly, since the introduction of Fully Convolutional Networks (FCN) (Shelhamer et al., 2017), which can produce probability prediction maps of the same size as the original images without restricting input image size, there has been significant progress in the image segmentation task. One of the most classical models in medical image segmentation task is UNet (Ronneberger et al., 2015). UNet combines lower-level detailed information with higher-level semantic information through its encoder-decoder structure and skip connections. Building upon UNet, Bauer et al. (2013) replaced the convolutional blocks in UNet with dense blocks Huang et al. (2017), enabling features reuse across channel dimensions, and leading to a more accurate and easily trainable network. Zhou et al. (2020) proposed the UNet++, which enhances the skip connection structure to aggregate features from various scales in the decoder sub-network, thereby improving model flexibility.

As medical images like MRI are often three-dimensional, one approach involves slicing the images into 2D slices along specific dimensions, training 2D models, and then reassembling the segmentation results into a 3D format (Tseng et al., 2017). Aslani et al. (2019) designed an end-to-end encoder-decoder network. They divided MRI images of MS patients into 2D slices along three dimensions, inputting them into multiple 2D segmentation models, and reassembled the resulting 2D segmentations into a 3D format using a majority voting approach. Zhang et al. (2019) proposed a method using 3D stacked slices that combine information from adjacent slices in multiple channels, increasing inter-slice information. To better extract inter-slice information, Çiçek et al. (2016) replaced 2D convolutional operations with 3D convolutions, fully utilizing information within and between image slices. Building upon this, La Rosa et al. (2020) proposed 3D UNet-, segmenting cortical and white matter lesions based on FLAIR and MP2RAGE sequences of MS patients. Hu et al. (2020) introduced a three-dimensional context-guided module in the encoding and decoding stages of 3D UNet, expanding the perceptual field, guiding contextual information, and enriching feature representations of MS lesion segmentation using a three-dimensional spatial attention block. Gessert et al. (2020) proposed a dual-path 3D convolutional structure with attention-guided interaction, separately processing MS data from two-time points and effectively exchanging information.

Due to the similarities between MS and NMOSD, classifying MS and NMOSD is also a critical step in auxiliary diagnosis.

Imaging-based classification methods using handcrafted features involve constructing a feature set from digital medical images and subsequently employing machine learning models for analysis. These methods typically require experienced radiologists to manually extract high-dimensional image data into low-dimensional handcrafted features. These features, along with relevant clinical variables, are used to create a feature set. Feature selection is then performed, and an optimal subset of features is utilized to build a predictive model. Huang (2019) extracted 273 radiomic features from the lesion area of patients’ brain T2-weighted images, including semantic, intensity, and texture features. They incorporated 11 radiomic features using the LASSO method, combined with 5 clinical features, to construct a diagnostic radiomic signature, achieving an AUC result of 0.93 on the test set. Kister et al. (2013) conducted quantitative analysis on the shape and distribution of localized T2 white matter lesions based on clinical brain MRI sequences of 44 AQP4-IgG antibody-positive NMOSD patients and 50 MS patients, creating a diagnostic procedure for classifying MS and NMOSD. Liu et al. (2019) extracted 9 features, including lesion heterogeneity and lesion volume, from patient imaging data, combined with clinical information, to build a logistic regression model to differentiate MS and NMOSD. However, these imaging-based methods rely heavily on radiologists manually extracting imaging features, limiting the repeatability and generalizability of these methods.

Deep learning-based classification methods efficiently capture classification features automatically without requiring manual extraction and selection. For instance, Hagiwara et al. (2021) developed an automatic classification model based on Convolutional Neural Networks (CNN). Due to limited available data, they primarily utilized SqueezeNet to prevent overfitting, achieving an accuracy of 0.81, sensitivity of 0.80, and specificity of 0.83 using common features to classify MS and NMOSD. Wang et al. (2020) compressed 3D MS and NMOSD MRI images into multi-channel 2D images and used a 2D ResNet model for classification. By leveraging transfer learning subsequent to pre-training the model on ImageNet, they attained an accuracy of 0.75. Kim et al. (2020) developed a 3D CNN model based on the ResNeXt concept to differentiate MS and NMOSD, which effectively utilized MRI spatial features and achieved improved performance with an accuracy of 0.71, sensitivity of 0.87, and specificity of 0.61 when integrating clinical information.

From existing research, it is evident that deep learning-based methods generally exhibit promising results in the task of classifying MS and NMOSD, often without extensive involvement from radiologists, thus possessing considerable practical value.

## 3 Materials and methods

### 3.1 Datasets and evaluation metrics

#### 3.1.1 Datasets

The datasets used in this study are MS and NMOSD MRI datasets. The MS datasets come from the Multiple Sclerosis Lesion Segmentation Challenge organized by the 2015 IEEE International Symposium on Biomedical Imaging (ISBI) (referred to as the ISBI dataset) and The First Hospital of Jilin University. The NMOSD dataset comes from the First Hospital of Jilin University.

Table 1 outlines the composition of the MS and NMOSD datasets utilized in this study. The ISBI dataset contains brain MRI images of 5 MS patients scanned at different time points, among which 4 patients scanned 4 groups of images and 1 patient scanned 5 groups of images, a total of 21 groups of image data, each group of images can utilize as a separate sample. Each group of images includes MRI images of four modalities: T1WI, T2WI, PD, and T2-FLAIR. This study only utilized the data of the T2-FLAIR modality. All the MRI images were scanned using a 3.0 Tesla MRI scanner and were registered to standard space, with the image size normalized to 181 × 217 × 181. At the same time, the ISBI dataset provides a lesion segmentation map manually annotated by experts corresponding to each image.

**Table 1:** MS and NMOSD datasets.

Disease classification	Data sources	Number of samples	Sequence type
MS	ISBI 2015	21	T2-Flair
	The First Hospital of Jilin University	48	T2-Flair
NMOSD	The First Hospital of Jilin University	62	T2-Flair

The brain MRI images of T2-FLAIR sequences obtained from the First Hospital of Jilin University comprised 48 MS samples and 62 NMOSD samples. Among them, all MS samples met the McDonald diagnostic criteria revised in 2017, and all NMOSD patients met the NMO diagnostic criteria revised in 2015. Due to the long collection time of the datasets, there are some differences in the collection parameters among images, as shown in Table 2.

**Table 2:** MRI parameters were collected from the First Hospital of Jilin University.

Parameter type	Parameter values
Slice number	160–192
Slice thickness (mm)	1
Repeat time (ms)	4,800
Echo time (ms)	279–324
Reverse time (ms)	1,650
Flip angle (°)	40

At the same time, the First Hospital of Jilin University provided lesion annotation maps for all images that were manually annotated by two radiologists with 5 and 10 years of experience in diagnosing brain diseases respectively, and were finally combined with the annotations of the two doctors. The intersection of the results is utilized as the annotation map of this study. Therefore, the datasets utilized in this study are a total of 69 MS samples and 62 NMOSD samples, all of which are T2-FLAIR modality.

#### 3.1.2 Data preprocessing

The ISBI dataset provides preprocessed images, which have been skull-stripped and registered to the MNI template. Therefore, we preprocessed the datasets provided by the First Hospital of Jilin University. The specific preprocessing steps are divided into the following four stages: Firstly, skull stripping was performed on the brain MRI images using the Brain Extraction Tool (BET) (Smith, 2002). Secondly, the images were corrected for bias field using the N4 bias field correction method (Tustison et al., 2010). Thirdly, the black background area of the images was removed. Finally, the size of the images is normalized to (160, 160, 160), while the voxel values of the images are normalized to a standard data distribution with a mean of 0 and a standard deviation of 1 using the Z-Score method.

#### 3.1.3 Data augmentation

**Figure 2:**
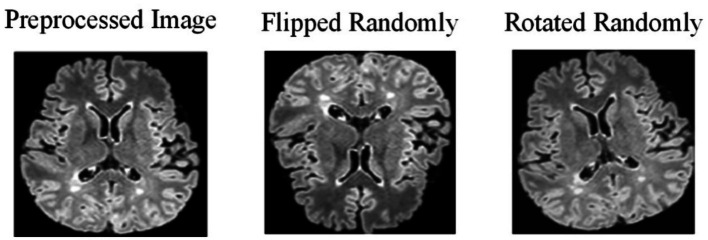
Effect of data augmentation.

Due to the challenges in collecting and annotating MS and NMOSD datasets, there is a limited amount of data available, making it difficult to train models. Therefore, this study utilizes data augmentation to expand the datasets and improve the generalization ability of the models. When training the deep learning models, the preprocessing images are flipped randomly (randomly selecting one of the three axes to flip) and rotated randomly with a fixed probability *p* = 0.5 each time (Figure 2).

### 3.2 Joint model

#### 3.2.1 Model overall architecture

**Figure 3:**
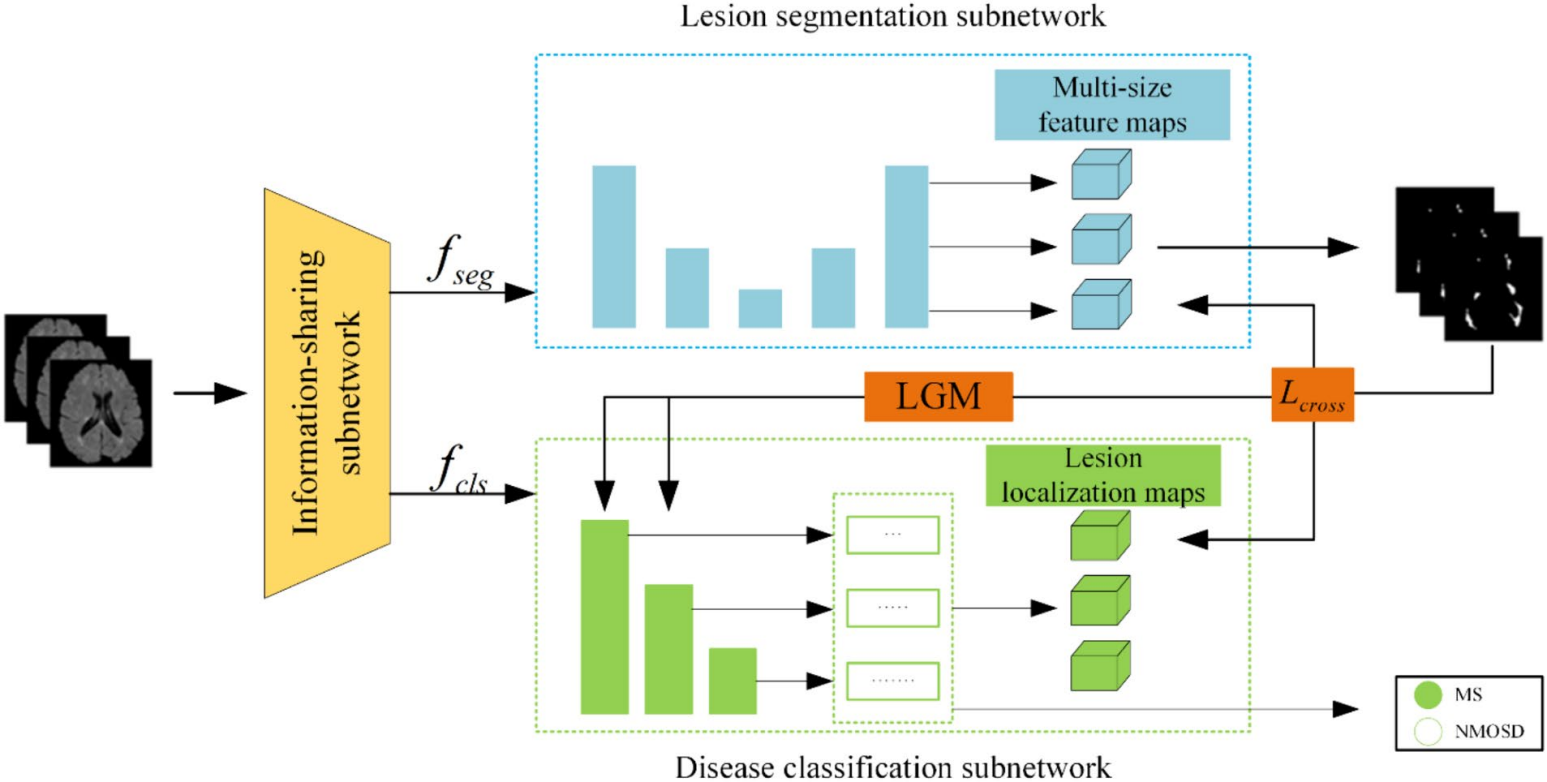
Structure of the joint model.

The joint model comprises three primary components: an information-sharing subnetwork, a lesion segmentation subnetwork, and a disease classification subnetwork. It leverages two types of information interaction methods to enhance the performance of both tasks. The overall architecture of this joint model is illustrated in Figure 3.

Initially, the MRI datasets are fed into the model and processed through the information-sharing subnetwork. Subsequently, the obtained segmentation feature maps $f_{seg}$ and classification feature maps $f_{cls}$ are inputted into the lesion segmentation subnetwork and the disease classification subnetwork, respectively. The outputs of the lesion segmentation subnetwork comprise binarized lesion segmentation maps, while the disease classification subnetwork provides classified predictions for MS or NMOSD.

Moreover, to bolster the interaction between these two tasks during the model training, we have introduced a Lesion Guidance Module (LGM) and a cross-task loss function. These additions aim to mutually guide and enhance the model’s performance.

#### 3.2.2 Information-sharing subnetwork

MS and NMOSD manifest characteristics of dispersion and multifocality, with lesions varying in shape, size, and discrete distribution. In the tasks involving segmentation and disease classification, it becomes imperative to consider not only the local details such as lesion shape and contour but also their global distribution within the brain. While convolutional operations primarily capture local information, they fail to establish long-distance dependencies across the entire image (He et al., 2016).

**Figure 4:**
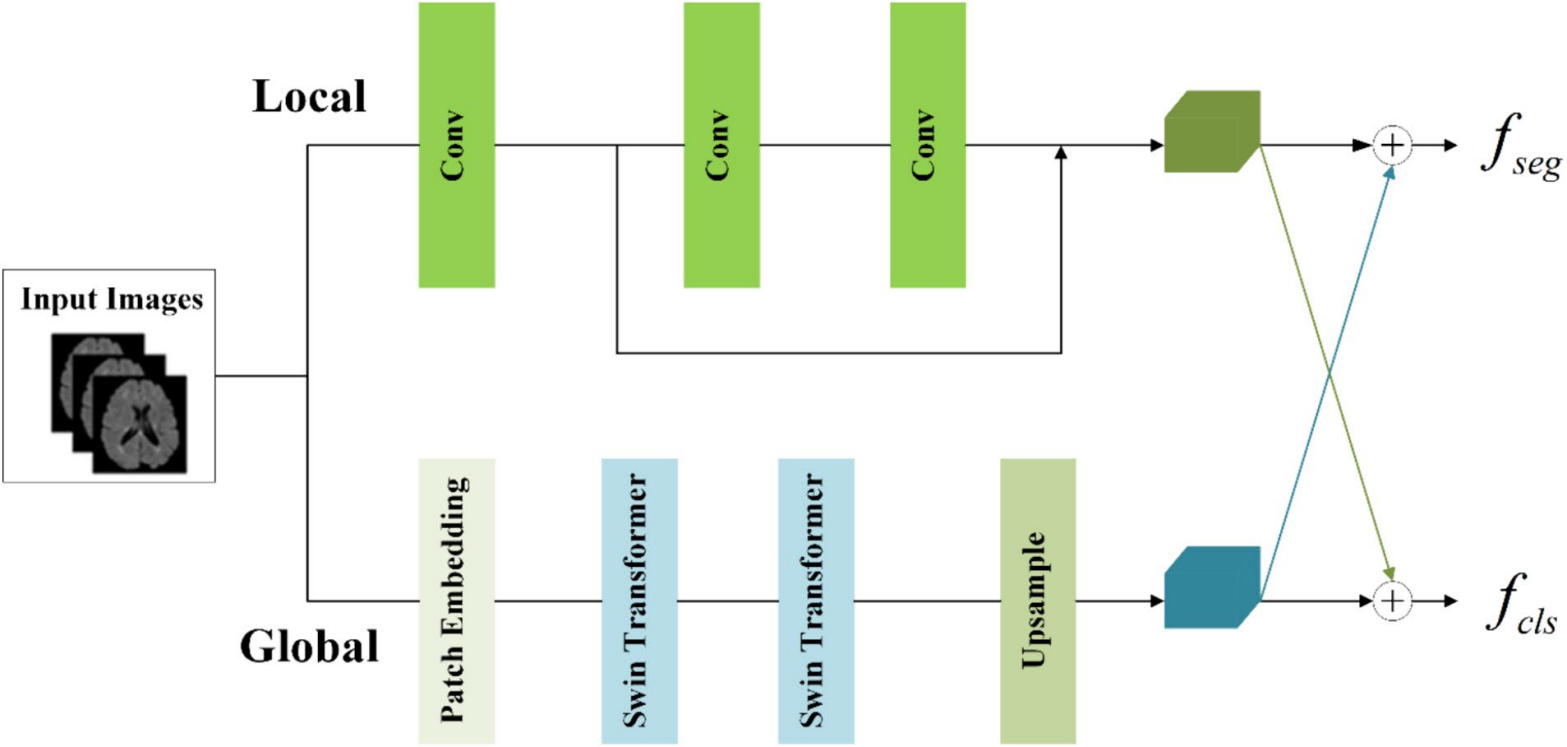
Structure of information-sharing subnetwork.

To concurrently capture both local and global information, the information-sharing subnetwork in the joint model is structured as a two-branch architecture. The local branch employs convolutional operations to extract detailed information about specific lesions, while the global branch utilizes Swin Transformer (Vaswani et al., 2017; Liu et al., 2021) coding modules to model long-range dependencies among image contexts. This specific architectural design is depicted in Figure 4.

To achieve the integration of local and global information, the segmentation feature maps for input into the segmentation subnetwork and classification feature maps for input into the classification subnetwork are obtained by combining them with cross-elements in a linear weighted manner.

In the global branch, to reduce computational complexity, image blocks of size 16 × 16 × 16 within the input images are treated as computational units for self-attention in the Transformer. A convolutional layer with a kernel size of 8 × 8 × 8 and a stride of 8 is utilized to extract features from the image $I\in {\mathbb{R}}^{H\times W\times D}$. This extraction process is followed by an average pooling operation, reducing the scale of the extracted features by half. The resulting feature maps $E\in {\mathbb{R}}^{\frac{H}{16}\times \frac{W}{16}\times \frac{D}{16}}$ serve as the input to the Transformer. The output of the global branch can be computed as follows (Equations 1,2):


(1) $$GTX_{\text{global}}=UP\left(ST\left(LN\left(E\right)\right)\right)$$

(2) E=PE(I)

Where PE(⋅) denotes patch embedding, which involves two operations: a convolution operation with a kernel size of 8 × 8 × 8 and a stride of 8, followed by an average pooling operation with a kernel size of 2 × 2 × 2 and a stride of 2. LN(⋅) represents layer normalization. ST(⋅) refers to two layers of Swin Transformer, with a window size of 5 × 5 × 5 and a switch between different window modes across these two layers (shift window mechanism). UP(⋅) denotes the output of the global branch achieved through interpolation. Through these operations, applied to the original images of size 160 × 160 × 160, we capture sufficient global information within this context.

In the local branch, three convolutional layers with 3 × 3 kernels are utilized as context extractors to capture local features. The first convolutional layer has a stride of 2, while the other two layers have a stride of 1. The output of the local branch can be computed as follows (Equation 3):


(3) $$CTX_{\text{local}}=\text{Conv}\left(LN\left(I\right)\right)$$

Where $CTX_{\text{local}}$ denotes the output of the local branch.

To enhance the information interaction between the classification and segmentation tasks, the local feature $CTX_{\text{local}}$ and global features $CTX_{\text{global}}$ are linearly combined by the crossover unit to obtain the segmentation feature maps $f_{\text{seg}}$ for the classification subnetwork and the classification feature maps $f_{\text{cls}}$ for the classification subnetwork respectively, which are calculated as Equation 4:


(4) $$\left[\begin{array}{l} f_{\text{seg}}\\ f_{\text{cls}} \end{array}\right]=[\begin{array}{l} w_{\text{seg},1}\\ w_{\text{seg},2} \end{array}\begin{array}{l} w_{\text{cls},2}\\ w_{\text{cls},1} \end{array}]\left[\begin{array}{l} CTX_{\text{local}}\\ CTX_{\text{global}} \end{array}\right]$$

Where $w_{\text{seg},1}$, $w_{\text{seg},2}$, $w_{\text{cls},1}$, and $w_{\text{cls},2}$ are learnable parameters. The size of $f_{\text{seg}}$ and $f_{\text{cls}}$ is both (32, 80, 80, 80).

#### 3.2.3 Lesion segmentation subnetwork

**Figure 5:**
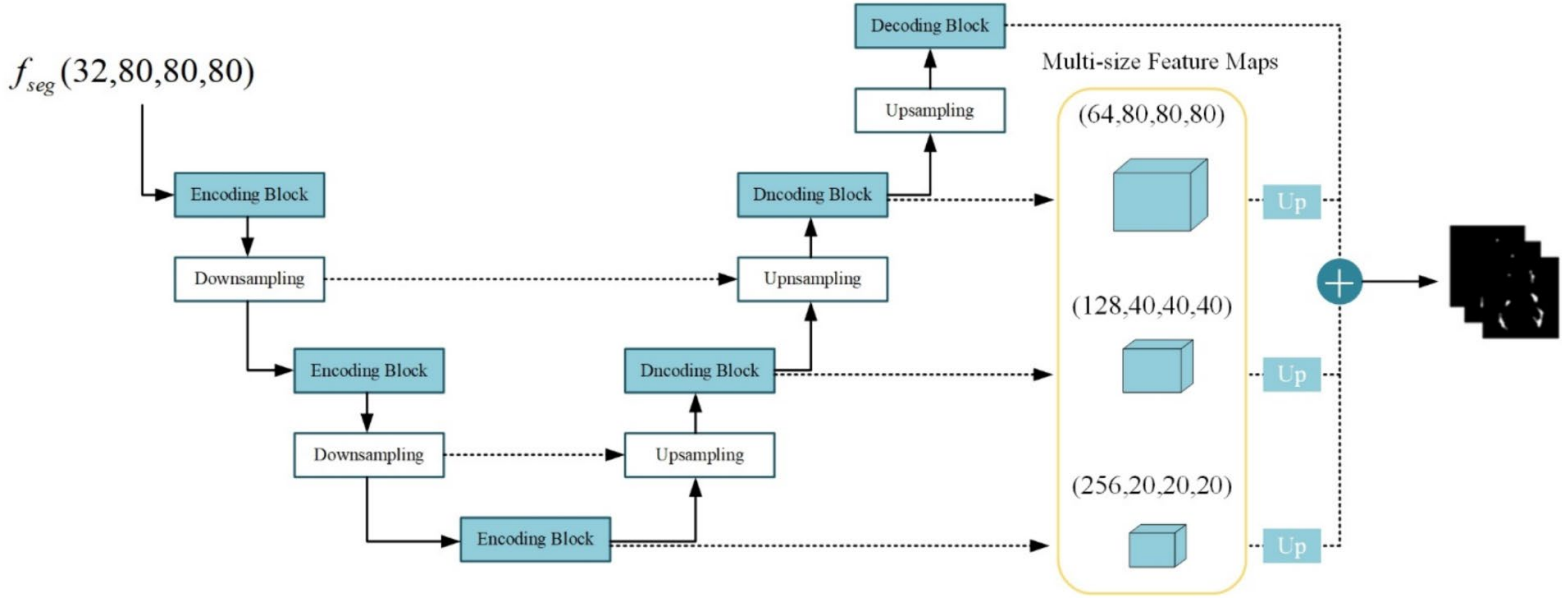
Structure of the lesion segmentation subnetwork.

The lesion segmentation subnetwork is utilized to segment brain white matter lesions in MS and NMOSD. Figure 5 shows the schematic structure of the lesion segmentation subnetwork, which mainly consists of three parts: the contraction path, the expansion path, and the multiscale binding module.

In the lesion segmentation subnetwork, the contraction path comprises three encoding modules and two downsampling modules aimed at capturing contextual information within the segmented feature maps $f_{\text{seg}}\in {\mathbb{R}}^{\frac{D}{2}\times \frac{H}{2}\times \frac{W}{2}}$ and extracting lesion-related features. Meanwhile, the expansion path involves three decoding blocks and three upsampling modules for restoring the feature maps to the dimensions of the input images. Between the contraction path and the expansion path, the low-level feature maps obtained from the contraction path and the high-level feature maps obtained from the expansion path are merged in the channel dimension through skip connections. This process aids in integrating detailed image information into the high-level semantic features, thereby enhancing segmentation performance. Additionally, the channel established by the skip connections between high and low levels facilitates gradient backpropagation (Zhang et al., 2018).

Given the substantial disparity in lesion sizes between MS and NMOSD, a strategy is employed to combine richer multi-scale features. Feature maps obtained at multiple levels are weighted and fused to generate the final lesion probability maps $M_{\text{lesion}}$ as follows (Equation 5):


(5) $$M_{\text{lession}}=\text{sigmoid}\left(\sum_{i=1}^{4}\left(\alpha_{i}\cdot Up\left(f_{1\times 1}\left(f_{\text{seg}}^{i}\right)\right)\right)\right)$$

Where $f_{1\times 1}\left(\cdot \right)$ denotes the convolution operation with a kernel size of 1 × 1 × 1 acting on the feature maps of size 20 × 20 × 20, 40 × 40 × 40, 80 × 80 × 80, 160 × 160 × 160 for adjusting the number of output channels to 1. Up(⋅) denotes the upsampling operation using nearest neighbor interpolation to resize the multi-scale feature maps to match the original image dimensions. Subsequently, the four-layer feature maps undergo a weighted combination with respective weights of 0.25, 0.25, 0.5, and 1. Finally, the feature maps are mapped to the (0, 1) interval using the sigmoid activation function, resulting in the final lesion probability maps.

#### 3.2.4 Disease classification subnetwork

**Figure 6:**
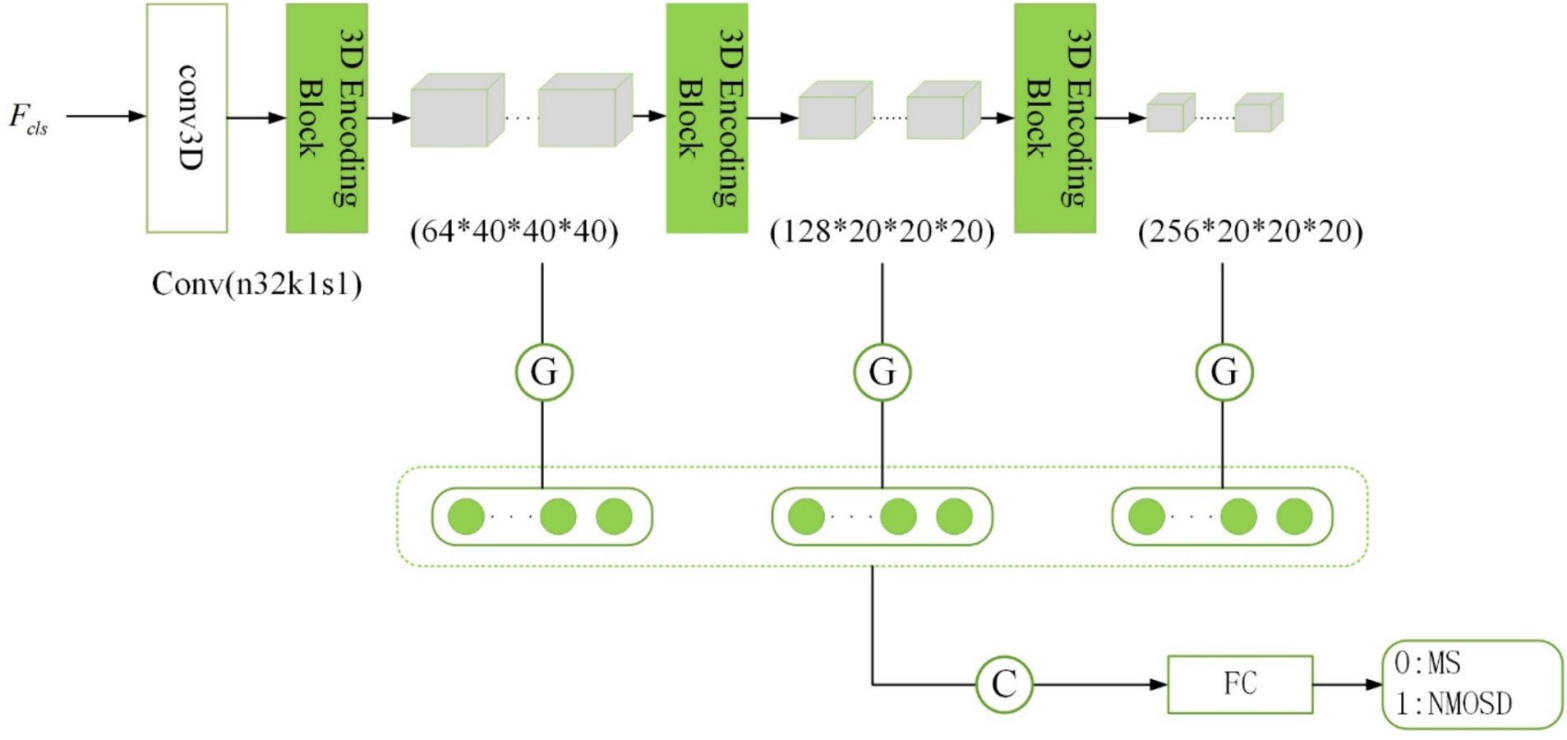
Structure of disease classification subnetwork.

The disease classification subnetwork is dedicated to the classification and diagnosis of two diseases, MS and NMOSD. The outputs $y^{\left(i\right)}={\left\{y^{\left(i\right)}\in \left\{0,1\right\}\right\}}_{i=1}^{N_{L}}$ provide classified predictions for MS or NMOSD. For the ith sample, $y^{\left(i\right)}=0$ denotes classification as MS, while $y^{\left(i\right)}=1$ denotes classification as NMOSD. Considering the relatively limited number of samples, an excessively complex or deeply layered model can lead to overfitting. In this study, the classification model is composed of three 3D coding blocks with residual connections, as depicted in the structure outlined in Figure 6.

The traditional approach in image classification involves unwinding the feature maps generated by the convolutional layer to form feature vectors for inputting into the fully connected layer. However, this method escalates the model’s parameter count, raising the risk of overfitting (Szegedy et al., 2015). In this study, we adopt a global average pooling of feature maps as an alternative to feature maps unwinding. There are several reasons for this approach: Firstly, global average pooling integrates spatial information from the feature maps, thereby enhancing the model’s generalization while preserving classification performance. Secondly, global average pooling requires fewer training parameters, mitigating the risk of overfitting associated with a fully connected layer.

Inspired by the concept of skip connections in the segmentation subnetwork, our model dynamically extracts classification features. The initial layers in the network capture low-level features like texture and color, while deeper layers extract high-level semantic features. To enhance the model’s performance, we combine the low-level feature maps with the high-level ones. Consequently, the final category prediction probability is computed as follows (Equation 6):


(6) $$p_{\text{cls}}=\text{soft}\max\left(f_{FC}\left(\sum_{k=1}^{64}f_{1,k\cdot w_{1}^{k,n}}+\sum_{k=2}^{128}f_{2,k}\cdot w_{2}^{k,n}+\sum_{k=3}^{256}f_{3,k\cdot w_{3}^{k,n}}\right)\right)$$

Where ${\left\{f_{1,k}\right\}}_{k=1}^{64}$, ${\left\{f_{2,k}\right\}}_{k=2}^{128}$, and ${\left\{f_{3,k}\right\}}_{k=3}^{256}$ denotes the feature vectors obtained after global average pooling of the feature maps of the three layers with different scales. These feature vectors were connected as the input of the fully connected layer $f_{FC}\left(\cdot \right)$ and then the softmax activation function was applied to obtain the classification prediction probabilities of MS and NMOSD.

### 3.3 Information interaction module

#### 3.3.1 Lesion guidance module

Since the lesion regions only occupy a small portion of the brain MRI, the majority of the image consists of normal brain tissues or blood vessels. This can present challenges for the model when attempting to accurately classify based solely on the presence of lesions. To address this, the lesion segmentation feature maps are utilized as prior information for lesion location and morphology by sharing the lesion probability maps obtained from the segmentation subnetwork into the classification subnetwork. By doing so, the influence of other parts of the MRI image on classification is mitigated, thereby facilitating disease classification and diagnosis.

**Figure 7:**
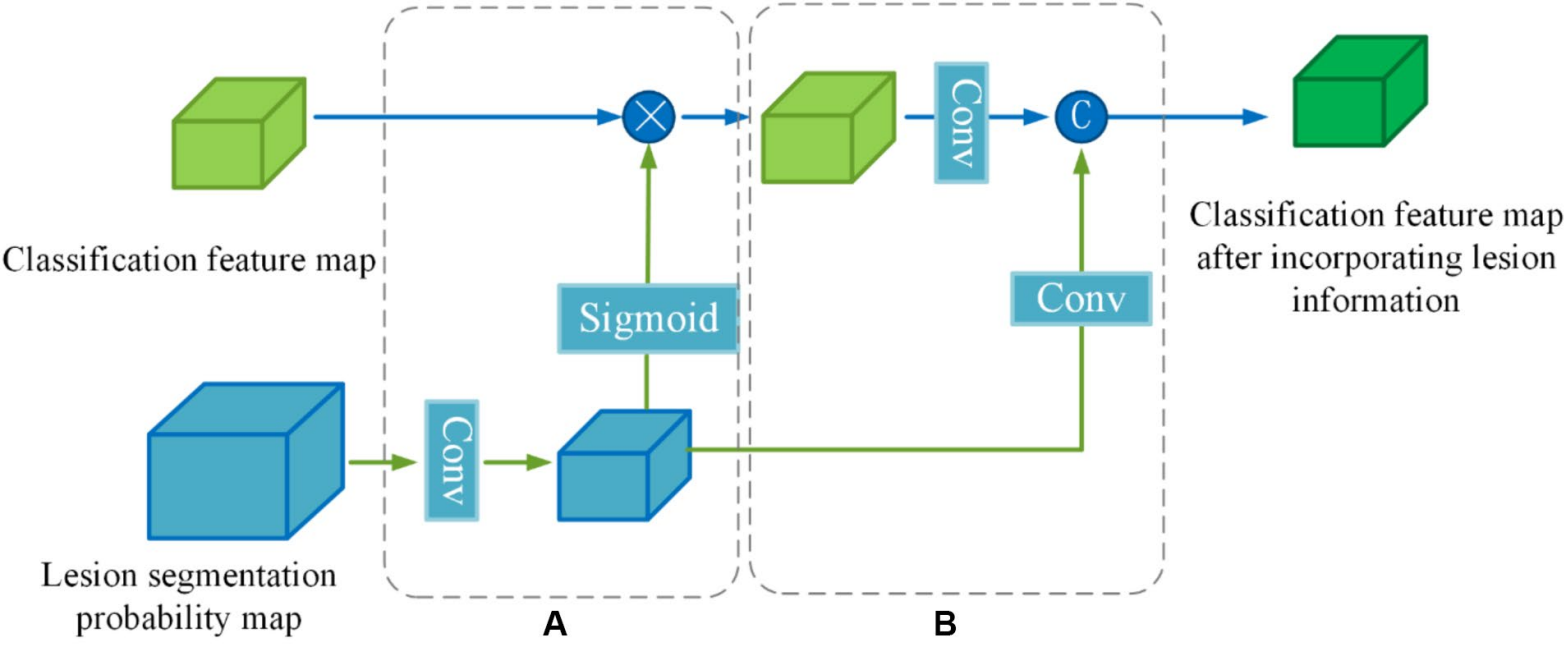
Structure of lesion guidance module.

To enhance the effectiveness of the segmented prior information, a Lesion Guidance Module (LGM) is proposed. The structure of the LGM, depicted in Figure 7, consists of two main components: computation of attention maps and fusion of raw features, corresponding to (a) and (b) in Figure 7, respectively.

Firstly, the lesion probability map, which is the output of the last layer of the segmentation subnetwork, undergoes feature transformation and normalization to generate the lesion attention map. Next, the lesion attention map is element-wise multiplied by the classification feature map, emphasizing the presence of lesions. The specific calculation process is as follows:

First, we utilize a 3 × 3 convolution with a stride of 2 to make the lesion segmentation probability map $\left\{P\right\}\in {\mathbb{R}}^{D\times W\times H}$ output from the last layer of the segmentation subnetwork to downsample to the same size as the categorized feature map $\left\{D,P^{\prime }\right\}\in {\mathbb{R}}^{\frac{D}{2}\times \frac{H}{2}\times \frac{W}{2}}$. Then the features are decoupled by a linear transformation of the Sigmoid function to generate the lesion attention map $\alpha_{i}$ (Equations 7,8):


(7) $$\alpha_{i}=\text{Soft}\max\left(f_{P}\left(P_{i}\right)\right)$$

(8) $$D^{\prime }=\alpha_{i}\cdot D$$

Where $\left\{P\right\}\in {\mathbb{R}}^{D\times W\times H}$ denotes the lesion segmentation probability map, $f_{P}\left(\cdot \right)$ denotes a convolution operation with a kernel size of 3 × 3 × 3 and a stride of 2, $\alpha_{i}\in {\mathbb{R}}^{\frac{D}{2}\times \frac{H}{2}\times \frac{W}{2}}$ denotes the lesion attention map, $D\in {\mathbb{R}}^{\frac{D}{2}\times \frac{H}{2}\times \frac{W}{2}}$ denotes classification feature map output from the information-sharing subnetwork, $D^{\prime }$ denotes segmentation feature map after emphasizing the lesions.

In order to further utilize the prior knowledge of lesion segmentation, the segmentation probability map is directly fused with the classification feature map after emphasizing the lesion in the channel dimension as a form of auxiliary information in the original feature fusion part. The joint features $\left\{J\right\}\in {\mathbb{R}}^{C\times \frac{D}{2}\times \frac{H}{2}\times \frac{W}{2}}$ are obtained by downscaling (Equation 9):


(9) $$J_{i}=f_{1\times 1}\left(\text{concat}\left(f_{3\times 3}\left(P_{i}^{’}\right),f_{3\times 3}\left(D_{i}^{’}\right)\right)\right)$$

Where $f_{1\times 1}\left(\cdot \right)$ denotes a convolution operation with a kernel size of 1 × 1 × 1,$f_{3\times 3}\left(\cdot \right)$ denotes a convolution operation with a kernel size of 3 × 3 × 3 and a stride of 2, $P_{i}^{\prime }$ denotes lesion segmentation probability map after downsampling, and concat(⋅) denotes channel dimension splicing.

#### 3.3.2 Cross-task loss function

For the classification task of MS and NMOSD, the basis for classification lies in the morphological and positional features of the lesions. Thus, the lesion point of the classification subnetwork during the classification process should be the lesion regions. Based on this analysis, this study utilizes the idea of CAM (Zhou et al., 2016) to achieve lesion localization in MRI images and generate lesion localization maps. To effectively assist the segmentation task, a cross-task loss function is proposed, which supervises the lesion localization map created in the classification subnetwork and the corresponding lesion segmentation maps in the segmentation subnetwork through a loss function. This helps enhance the lesion localization ability in each task. According to the theory of CAM, the lesion localization maps are obtained by global average pooling the feature maps, multiplying the resulting scalar with the class weights corresponding to the output layer, and accumulating it with the feature maps. To incorporate multi-scale features, this study generates lesion localization maps at three different layers. The specific calculation formula is as follows (Equation 10):


(10) $$V_{i}=\sum_{k=1}^{N}F_{i,k}\cdot w_{i,k}^{c}$$

Where $V_{i}$ represents the lesion localization map formed in the ith layer (i = 1,2,3), N represents the total number of feature maps in the ith layer, $F_{j,k}$ represents the kth feature map in the ith layer, and $w_{i,k}^{c}$ represents the weight corresponding to class c. Then, the lesion localization maps are weighted and computed with the lesion segmentation maps of the same size from the segmentation subnetwork using the Mean Square Error (MSE) loss. This results in a cross-task loss function, with the specific formula as follows (Equation 11):


(11) $$L_{\text{cross}}=\sum_{i=1}^{3}\eta_{i}\cdot \left\|S_{i}-V_{i}{\;}_{2}^{2}\right\|$$

Where $S_{i}$ represents the intermediate multi-size segmentation maps obtained from the lesion segmentation subnetwork. $\eta_{i}$ are set to 0.25, 0.25, and 0.5, respectively.

Furthermore, the generation of lesion localization maps achieves the interpretability of deep learning black-box models in the process of classification diagnosis, which is of significant importance for research related to medically auxiliary diagnosis.

### 3.4 Overall loss function

For the disease classification task, the difference between the predicted categories and the true labels was evaluated using the binary cross entropy loss with the formula Equation 12:


(12) $$L_{\text{cls}}=-\sum y\text{log}\hat{y}+\left(1-y\right)\text{log}\left(1-\hat{y}\right)$$

Where $\hat{y}$ represents the categories of model predictions, y represents the real categories.

For the lesion segmentation task, the Dice Similarity Coefficient (DSC) is utilized to measure the degree of similarity between the segmentation results and the real segmentation maps, which can measure the accuracy of the segmentation results and take the value in the range of [0,1]. Therefore, the segmentation model can be supervised by using 1-Dice as a loss function, called Dice loss, the specific formula is Equation 13:


(13) $$L_{\text{seg}}=1-\text{Dice}=1-\frac{2\left|X\cap Y\right|}{\left|X\right|+\left|Y\right|}$$

Where X represents the segmentation result of the lesion segmentation task, Y represents the real segmentation result, and |⋅| represents the number of voxels that satisfy the condition.

Therefore, to optimize the learnable parameters $w_{t}=\left(\theta_{t}\right)$ of the joint model, where $\theta_{t}$ is the model parameter, an overall loss function for the joint model is designed in conjunction with the single-task loss described above. Since single tasks may have different levels of contribution in optimizing the parameters of the model, the single task loss function is weighted by setting a weighting factor $\beta_{t}$, and the joint loss function constituted is Equation 14:


(14) $$L=\beta_{1}L_{\text{seg}}+\beta_{2}L_{\text{cls}}+\beta_{3}L_{\text{cross}}$$

The variable $\beta_{i},i=1,2,3$ represents a hyperparameter. Considering that the segmentation task involves pixel-level classification of images, while the classification task involves categorizing individual samples, segmentation tasks are comparatively more complex and challenging to learn. Therefore, in the training process, the contribution of parameter optimization for the segmentation task should be relatively higher. Hence, in this study, $\beta_{1}$, $\beta_{2}$, and $\beta_{3}$ are set to 1, 0.8, and 1, respectively.

## 4 Experiment and results

### 4.1 Experiment settings

The hardware platform for the experiments in this study is the NAVIDA GTX 3090 graphics card and the network models are all built by the PyTorch framework. During model training, the batch size was set to 2, the number of iterations was set to 200, the optimization algorithm utilized Adam optimizer with default parameters (Kingma and Ba, 2014), and the learning rate was initially set to 0.0001. If the loss of the model did not decrease after surpassing 10 training iterations, the learning rate was then reduced to half of its original value.

To reduce experimental variability and provide a more accurate and objective reflection of model performance, we utilized a five-fold cross-validation strategy during the experiment. Initially, the entire dataset is randomly divided into five subsets. During each training iteration, four of these subsets are used as the training set, while the remaining subset serves as the test set. Upon completion of each training iteration, evaluation results are obtained on the corresponding test set. The final experimental outcome is determined by averaging the evaluation results obtained from the five training iterations.

The images used in the experiments are obtained from the dataset after undergoing the data preprocessing and data augmentation described in Section 3.1. The original size of each input is (160, 160, 160). After being processed by the information-sharing subnetwork, the inputs for the lesion segmentation subnetwork and disease classification subnetwork are resized to (80, 80, 80).

### 4.2 Evaluation metrics

The joint model proposed in this study mainly consists of two tasks, the lesion segmentation task and the disease classification task. Multiple evaluation metrics were employed to assess the performance of each model.

The evaluation metrics utilized for the segmentation task are Dice Similarity Coefficient (DSC), Positive Predict Value (PPV), True Positive Rate (TPR), and Volume Difference (VD) (Equations 15–18).


(15) $$DSC=\frac{2\times \left|Y\cap \hat{Y}\right|}{\left|Y\right|+\left|\hat{Y}\right|}$$

Where Y represents the ground truth, $\hat{Y}$ represents the output of the model, ∩ represents the intersection operation of two matrices, and |.| represents the number of elements in the matrix. The higher the DSC, the closer the prediction to the manually segmented label.


(16) $$PPV=\frac{\left|Y\cap \hat{Y}\right|}{\left|\hat{Y}\right|}$$

PPV represents the proportion of true positive voxels among all voxels predicted as positive. In this context, positive refers to the lesion voxels. A higher PPV indicates that the impact of the noise caused by them on the model is smaller.


(17) $$TPR=\frac{\left|Y\cap \hat{Y}\right|}{\left|Y\right|}$$

TPR represents the proportion of true positive voxels among all actual positive voxels. The higher the TPR, the stronger the model’s ability to identify lesions.


(18) $$VD=\frac{TP_{P}-TP_{gt}}{TP_{gt}}$$

Where $TP_{p}$ represents the number of predicted TP voxels and $TP_{gt}$ represents the number of lesion voxels in the ground truth. A lower VD indicates a better agreement between the predicted and true lesion volumes.

The metrics utilized in the classification task are Accuracy (ACC), Sensitivity (SN), Specificity (SP) (Equations 19–21), and Area Under the ROC Curve (AUC).


(19) $$ACC=\frac{TP+TN}{TP+FP+FN+FN}$$

(20) $$SN=\frac{TP}{TP+FN}$$

(21) $$SP=\frac{TN}{TN+FP}$$

Where TP represents true positive, TN represents true negative, FP represents false positive, and FN represents false negative. These metrics can be used to assess the performance of classification models.

### 4.3 Comparison and analysis of experimental results

To validate the superiority of the proposed joint model for lesion segmentation and disease classification in MS and NMOSD, this section conducts comparative analyses between the joint model and three advanced segmentation methods: 3D UNet, VNet, and AttentionUNet, as well as three advanced classification methods: 3D ResNet34, 3D ResNet50, and 3D DenseNet. Throughout the experiments, efforts are made to ensure that the primary parameters of all methods remain consistent with those introduced in Section 4.1.

#### 4.3.1 Comparison of the segmentation methods

Table 3 shows the comparison of the results of the joint model on lesion segmentation. It can be seen that the joint model performs the best on the lesion segmentation task and achieves the highest DSC, TPR, and VD, which are 74.87, 72.21, and 22.34%, respectively, and PPV achieves the sub-optimal results, which is second only to the 3D UNet. Where AttentionUNet achieves sub-optimal results on DSC, TPR, and VD which differed from the joint model by 2.8, 0.89, and 4.33%, respectively. The results prove that the lesion segmentation results obtained by the joint model have higher similarity with the real lesion segmentation results and higher check-accuracy for lesion pixel points. The result of PPV is lower than that of 3D UNet by 0.17%, proving that checking accuracy for lesion pixel points of the segmentation model is slightly lower than 3D UNet. Overall, the joint model has the optimal segmentation effect, and AttentionUNet is the second best.

**Table 3:** Comparative experimental results of the lesion segmentation method.

Method	DSC (%)	PPV (%)	TPR (%)	VD (%)
3D UNet	70.64	**74.11**	65.25	36.38
VNet	71.93	72.24	69.71	43.43
AttentionUNet	72.07	70.22	71.32	26.67
Joint Model	**74.87**	73.94	**72.21**	**22.34**

Bold values indicate the best results in evaluation metrics. Underlined values indicate the second best results in evaluation metrics.

**Figure 8:**
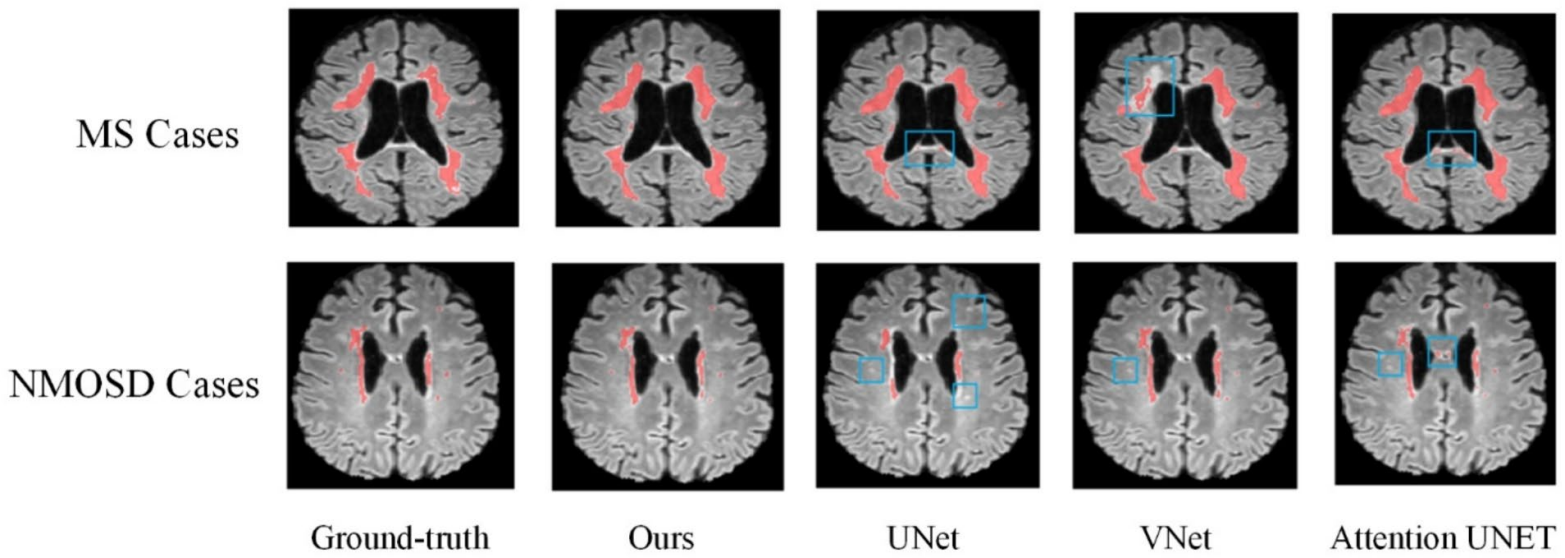
Comparison of experimental visualization results.

Figure 8 shows the lesion segmentation visualization results of the four segmentation models acting on a MS case and a NMOSD case, respectively. In order to clearly demonstrate the segmentation effect, the lesion segmentation results are superimposed on the original MRI image in red. From left to right, it shows the ground truth segmentation annotated manually by the doctors, the joint model segmentation results, the UNet segmentation results, the VNet segmentation results, and the AttentionUNet segmentation results. As can be seen from the visualization results, the models can achieve localization for lesion regions with relatively large volumes. However, the discrete point lesions in the brains of MS and NMOSD patients are difficult to recognize, as well as some regions in the brain MRI with similar imaging features to the white matter high signals are very easy to confuse during segmentation. The blue boxed part in the figure shows the under-segmentation or over-segmentation problem of the model during segmentation. In contrast, the joint model greatly avoids the above problems due to the combination of the lesion location information provided by the classification network, and the segmentation results are closer to the ground truth segmentation.

Overall, based on evaluations of various metrics and visualized results, it can be observed that the segmentation performance of the joint model is superior. Particularly for some challenging pinpoint lesions and locations with similar imaging characteristics, the segmentation results achieved by the joint model is more refined. Given the highly irregular morphology of MS and NMOSD lesions, even expert radiologists find it challenging to completely delineate their contours during annotation. However, the model proposed in our study ensures the ability to accurately locate the majority of lesions.

#### 4.3.2 Comparison of the classification methods

**Figure 9:**
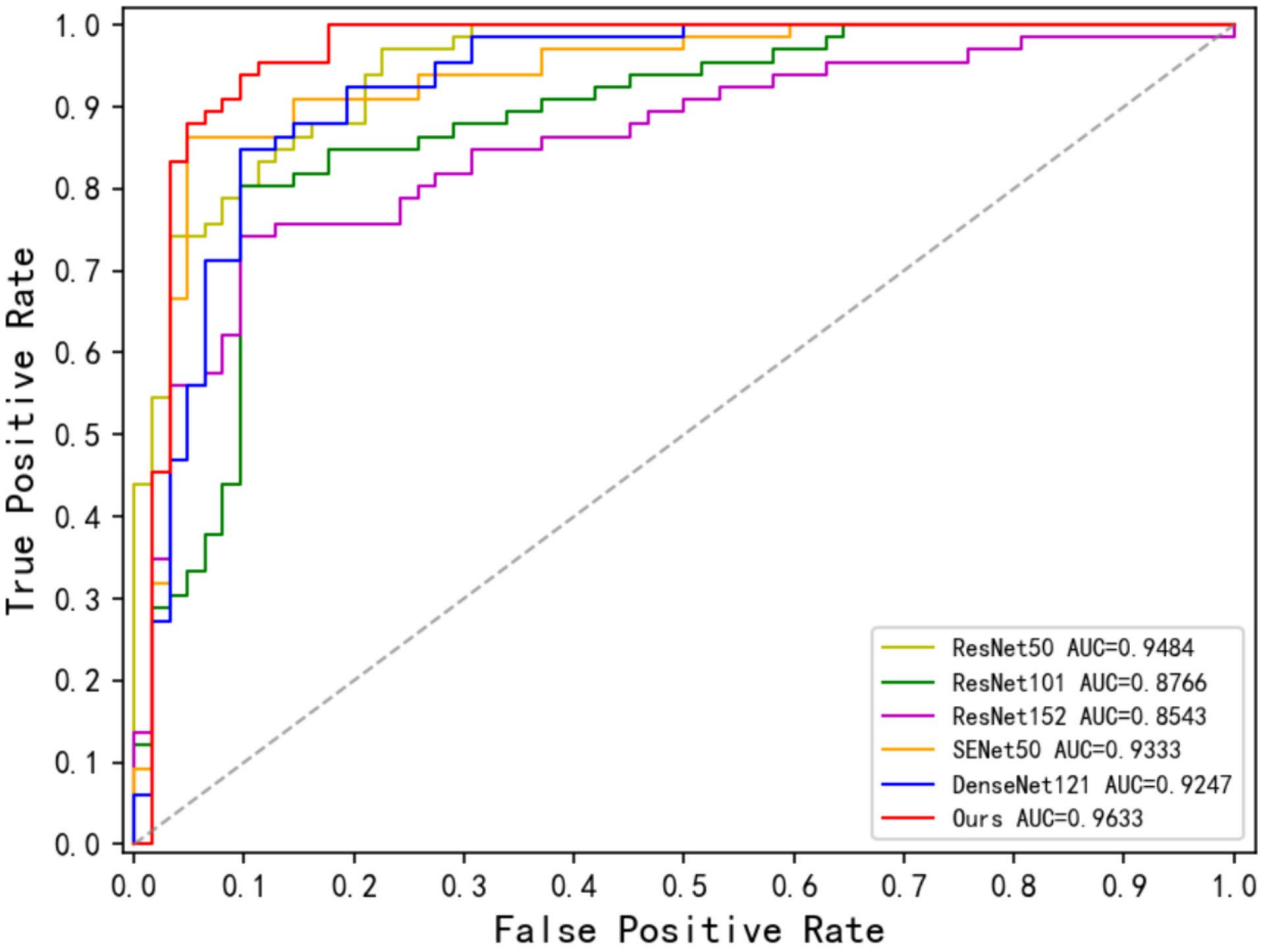
ROC curve of the classification methods.

Table 4 demonstrates the disease classification results of the joint model and the other five advanced classification models for MS and NMOSD. From the results in the table, it can be seen that the joint model achieved a classification accuracy of 92.16%, which is the best performance among several methods and 2.36% higher than SENet50 with the second-best accuracy. In addition, the ROC curves for the classification results of the five methods are shown in Figure 9. Compared with the other models, the joint model has the largest area under the line of the ROC curve, which is 96.33%, indicating that the joint model has a better effect.

**Table 4:** Comparative experimental results of lesion classification method.

Method	ACC (%)	SN (%)	SP (%)	AUC (%)
ResNet50	87.24	87.14	88.33	94.84
ResNet101	86.63	80.28	92.38	87.66
ResNet152	86.40	85.52	85.47	85.43
DenseNet121	88.36	90.85	83.57	92.47
SENet50	89.80	86.00	93.57	93.33
Joint Model	**92.16**	**95.60**	**92.60**	**96.33**

Bold values indicate the best results in evaluation metrics.

### 4.4 Results and analysis of ablation experiments

The joint model proposed in this study focuses on the mutual guidance of the lesion segmentation and disease classification tasks through an information-sharing subnetwork and two information interaction methods which include the lesion guidance module and the cross-task loss function. In this section, we will first validate the effectiveness of the joint learning strategy, and then conduct ablation experiments on the information sharing subnetwork and the two information interaction methods, respectively, to verify the influence of each component in improving the performance of the two tasks.

#### 4.4.1 Influence of the joint learning strategy

To demonstrate the impact of joint learning strategy on model performance, this section will compare the results of the joint model and two fundamental models: the lesion segmentation model (corresponding to the lesion segmentation subnetwork in Section 3.2.3) and the disease classification model (corresponding to the disease classification subnetwork in section 3.2.4). This comparison aims to establish the effectiveness of the joint learning strategy. The joint model will be abbreviated as the Joint Model, the lesion segmentation model will be abbreviated as Only Seg Model and the disease classification model will be abbreviated as Only Cls Model.

(1) Influence on the lesion segmentation task

Table 5 compares the performance of the Only Seg Model and Joint Model in lesion segmentation tasks. The Joint Model demonstrated improvement across all metrics compared to the Only Seg Model, with an increase of 3.63% in DSC, 3.80% in PPV, 2.34% in TPR, and 2.63% in VD. This demonstrates that incorporating information from the classification task effectively enhances the performance of lesion segmentation.

**Table 5:** The influence of the joint learning strategy on segmentation task.

Method	DSC (%)	PPV (%)	TPR (%)	VD (%)
Only Seg Model	71.24	70.14	69.87	24.97
Joint Model	**74.87**	**73.94**	**72.21**	**22.34**

Bold values indicate the best results in evaluation metrics.

**Figure 10:**
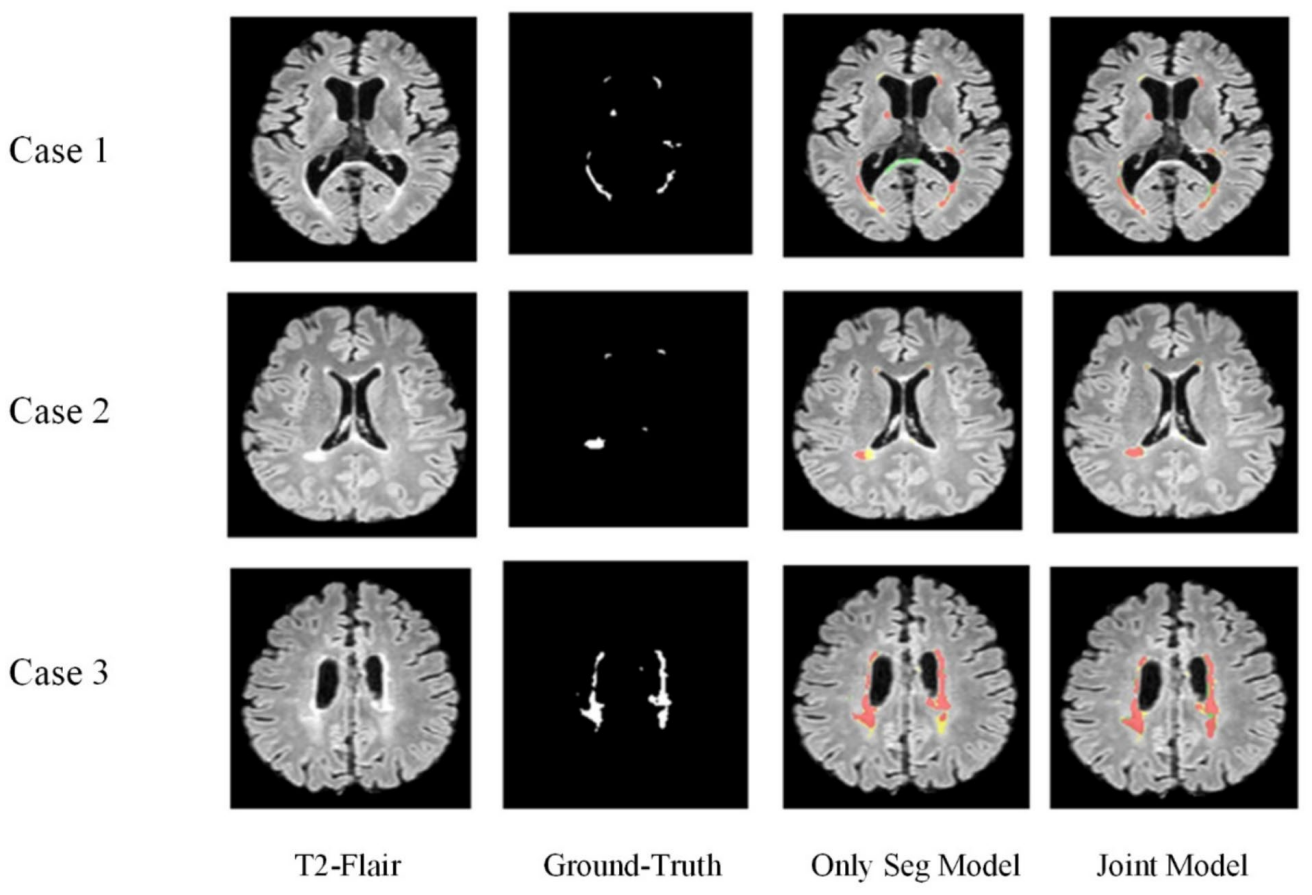
Visual display of lesion segmentation results.

Figure 10 displays the lesion segmentation results using the Only Seg Model and Joint Model for three different cases. From left to right: original T2-FLAIR images, manually segmented images, visualizations of results from the Only Seg Model, and visualizations of results from the Joint Model. In the visualizations, red indicates true positives—pixels classified correctly as lesions; green represents false positives—pixels classified as lesions but normal tissues; yellow denotes false negatives—pixels classified as normal tissues but lesions. In the segmentation results of Case One, the Only Seg Model misclassifies some normal tissue as lesions (shown in green) due to its similarity to high-intensity white matter, leading to misjudgments. However, the Joint Model correctly identifies this portion. For Case Two, the Only Seg Model struggles to delineate the contours of patchy lesions (depicted in yellow), indicating segmentation inadequacies. Similar issues of segmentation insufficiency are observed for Case Three with the Only Seg Model (yellow portion). Because lesions in MS and NMOSD often exhibit highly irregular shapes, segmenting lesion edges presents a challenge. The visual results show that both the Only Seg Model and Joint Model have some false positives and negatives along the lesion edges. Nevertheless, overall, the Joint Model demonstrates significantly better segmentation performance compared to the Only Seg Model.

The visual results demonstrate that the segmentation performance of the Joint Model surpasses that of the Only Seg Model across all four samples. This outcome suggests that the lesion features extracted from the classification task, especially positional characteristics, effectively assist the segmentation task in capturing the varied locations and sizes of MS and NMOSD lesions.

(2) Influence on the Disease Classification task

Table 6 is the comparison between the Only Cls Model and the Joint model on the results of the MS and NMOSD classification task. Compared to the Only Cls Model, the joint model demonstrated improvement across all metrics, with ACC increasing by 4.36%, SN by 8.36%, and SP by 3.14%.

**Table 6:** The influence of the joint learning strategy on classification task.

Method	ACC (%)	SN (%)	SP (%)	AUC (%)
Only Cls Model	87.80	87.24	89.46	87.59
Joint Model	**92.16**	**95.60**	**92.60**	**96.33**

Bold values indicate the best results in evaluation metrics.

**Figure 11:**
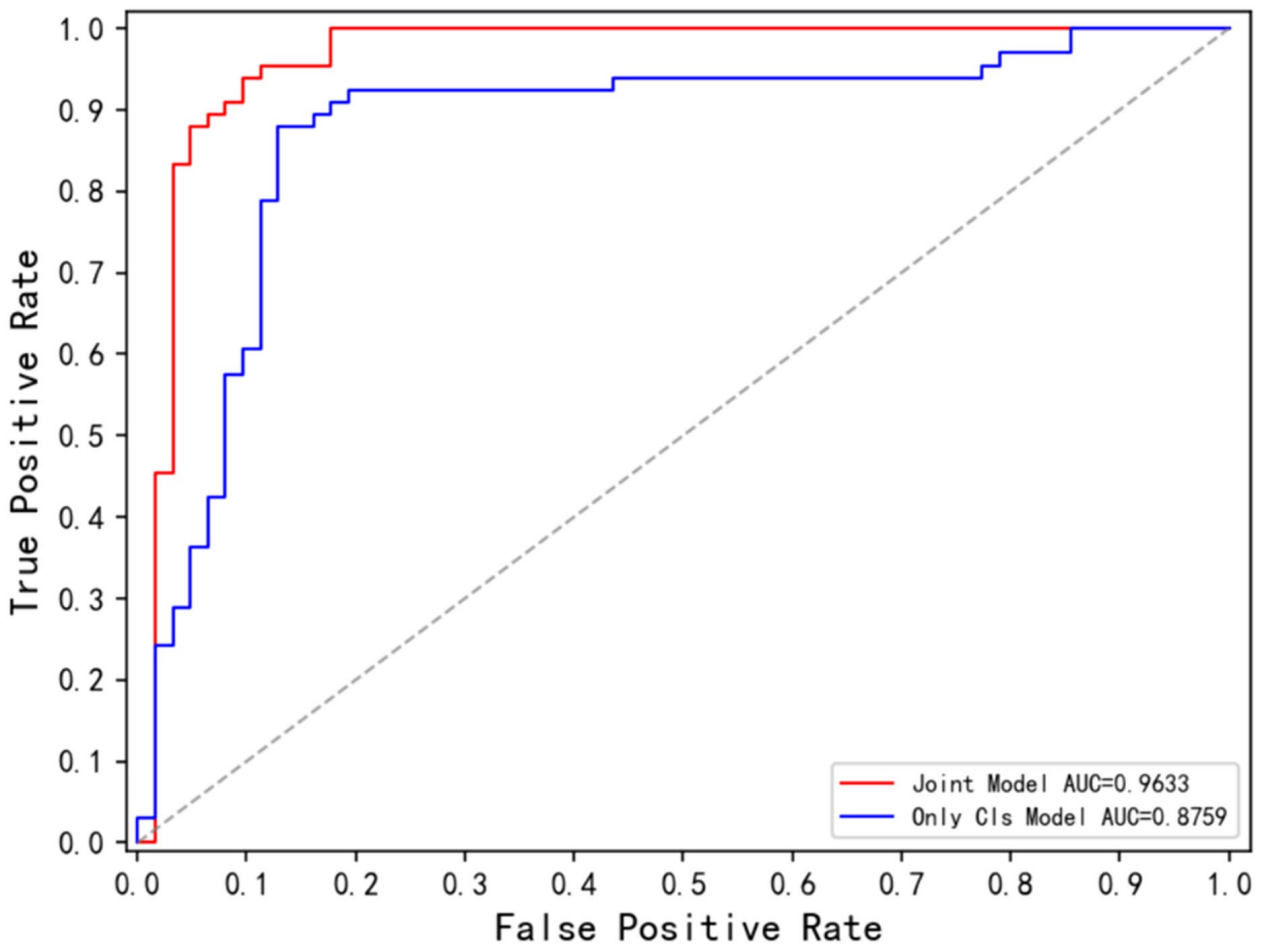
ROC curve corresponding to Joint Model and Only Cls Model.

Figure 11 displays the ROC curves for the Only Cls Model and Joint Model in the classification task. The AUC for the Joint Model is 96.33%, while the AUC for the Only Cls Model is 87.59%. When combined with the table, this demonstrates that leveraging the information extracted from the segmentation task and utilizing the segmented lesion results effectively guides the classification task, thereby enhancing the overall performance of the classification task.

Based on the comprehensive analysis, the Joint Model based on the joint learning strategy demonstrated performance improvements in both lesion segmentation and disease classification tasks. It exhibited superior results across various metrics and visualization outputs compared to single-task models. This validates that the joint learning strategy effectively leverages the features, harnesses hidden information learned from shared classification and segmentation tasks, and enhances the model’s fitting capability. Consequently, it elevates the performance in both tasks.

#### 4.4.2 Influence of the information sharing module

To investigate the effectiveness of the dual-branch structure within the information-sharing subnetwork, the following experiments were conducted in this section: Removing the information-sharing subnetwork, denoted as ‘w/o share’; Using only the local branch to share underlying information through hard parameter sharing, denoted as ‘only local’; Using only the global branch to share underlying information through hard parameter sharing, denoted as ‘only global.’

Table 7 presents the ablation experiment results for the information-sharing subnetwork. The experimental findings indicate that the information-sharing subnet significantly enhances the performance of both lesion segmentation and disease classification tasks. Performance is notably poorest when the information-sharing subnet is entirely removed, while the Joint Model demonstrates the optimal performance. When utilizing only the local branch for hard parameter sharing of the underlying information, the performance in the lesion segmentation task ranks second, following closely behind the Joint Model. This demonstrates that local lesion information, such as morphology and edges, holds greater significance for the lesion segmentation task. When employing local sharing, the model prioritizes aspects related to the segmentation task. In the case of using only the global branch for hard parameter sharing of the underlying information, the performance in the disease classification task ranks second, closely following the Joint Model. This highlights that global image information, such as lesion distribution, plays a more advantageous role in the disease classification task.

**Table 7:** The influence of the information-sharing module.

Method	DSC (%)	PPV (%)	TPR (%)	VD (%)	ACC (%)	SN (%)	SP (%)	AUC (%)
w/o share	71.98	72.11	67.47	26.35	88.20	86.24	89.24	88.56
Only local	73.80	73.95	71.34	24.12	86.14	86.00	88.26	89.32
Only global	72.04	70.18	70.44	24.92	90.22	91.46	89.60	92.68
Joint Model	**74.87**	73.94	**72.21**	**22.34**	92.16	**95.60**	**92.60**	**96.33**

Bold values indicate the best results in evaluation metrics. Underlined values indicate the second best results in evaluation metrics.

#### 4.4.3 Influence of the lesion guidance module

As mentioned in section 3.3, the information interaction consists of two parts, one is the LGM for combining the results of lesion segmentation, and the other is the cross-task loss function. In this section, the effectiveness of the lesion guidance module is verified through ablation experiments.

The LGM serves to utilize the segmentation probability maps as prior information about lesion distribution and morphology to guide the classification subnet. Its structure involves emphasizing lesions by first applying an attention mechanism through the dot product operation between the segmentation probability maps and the classification feature maps. Subsequently, the segmentation probability maps are concatenated with the classification feature maps along the channel dimension to further integrate lesion information. In this section, we investigate the effectiveness of LGM for the classification task, as well as the efficacy of the LGM structure. We conduct ablation experiments as follows: (1) Removing the LGM, denoted as ‘w/o LGM’. (2) Using only the dot product operation to combine the segmentation probability maps and the classification feature maps, denoted as ‘dot product’. (3) Using only the concatenation along the channel dimension to combine the segmentation probability maps and the classification feature maps, denoted as ‘concat.’ (4) Given the common approach of fusing information by pixel-wise addition, such as in, we compare using the addition operation to combine the segmentation probability maps and the classification feature maps, denoted as ‘dot add.’ Table 8 presents the performance of different forms of LGM on the classification task.

**Table 8:** The effect of the LGM on classification performance.

Interactive mode	ACC (%)	SN (%)	SP (%)	AUC (%)
w/o LGM	89.44	89.30	86.24	89.38
dot product	91.20	93.42	89.28	94.36
concat	90.66	93.34	91.60	92.24
dot add	89.48	89.36	85.24	89.90
Joint Model	**92.16**	**95.60**	**92.60**	**96.33**

Bold values indicate the best results in evaluation metrics. Underlined values indicate the second best results in evaluation metrics.

Table 8 highlights that removing the LGM notably worsens classification performance, underlining the importance of merging lesion segmentation into disease classification. Using attention on lesion segmentation probability maps, like with dot product, notably boosts classification, although it’s not the best method.

Combining the segmentation maps with classification features through channel dimension concatenation and dot product both improve the classification. However, channel concatenation works better, likely because the segmentation maps may be inaccurate, causing problems with direct addition or summation.

The proposed LGM, using both dot product and channel concatenation, significantly enhanced classification performance, offered the most improvement.

#### 4.4.4 Influence of cross-task loss function

We set up ablation experiments for the cross-task loss function of the information interaction approach in this section. According to Equation (14), the overall loss function of the model consists of three parts: segmentation loss function, categorization loss function, and cross-task loss function, which is verified in this section by removing the cross-task loss function, called w/o cross loss.

Table 9 demonstrates the impact of cross-task loss functions on lesion segmentation and disease classification tasks. The results indicate that cross-task loss functions effectively enhance the performance of both tasks, particularly in the case of lesion segmentation. The average metrics show an improvement of 1.23%, validating that the lesion localization maps constructed by the classification subnetwork effectively aid the segmentation subnetwork in localizing lesions, thereby enhancing segmentation performance. For the disease classification task, there are improvements across metrics such as ACC, SP, and AUC. This demonstrates that the feature maps obtained by the segmentation subnetwork effectively guide the classification subnet in capturing lesions.

**Table 9:** The effect of cross-task loss function.

Method	DSC (%)	PPV (%)	TPR (%)	VD (%)	ACC (%)	SN (%)	SP (%)	AUC (%)
w/o cross loss	73.96	72.06	70.64	22.90	92.08	**95.62**	92.54	95.54
Joint Model	**74.87**	**73.94**	**72.21**	**22.34**	**92.16**	95.60	**92.60**	**96.33**

Bold values indicate the best results in evaluation metrics. Underlined values indicate the second best results in evaluation metrics.

**Figure 12:**
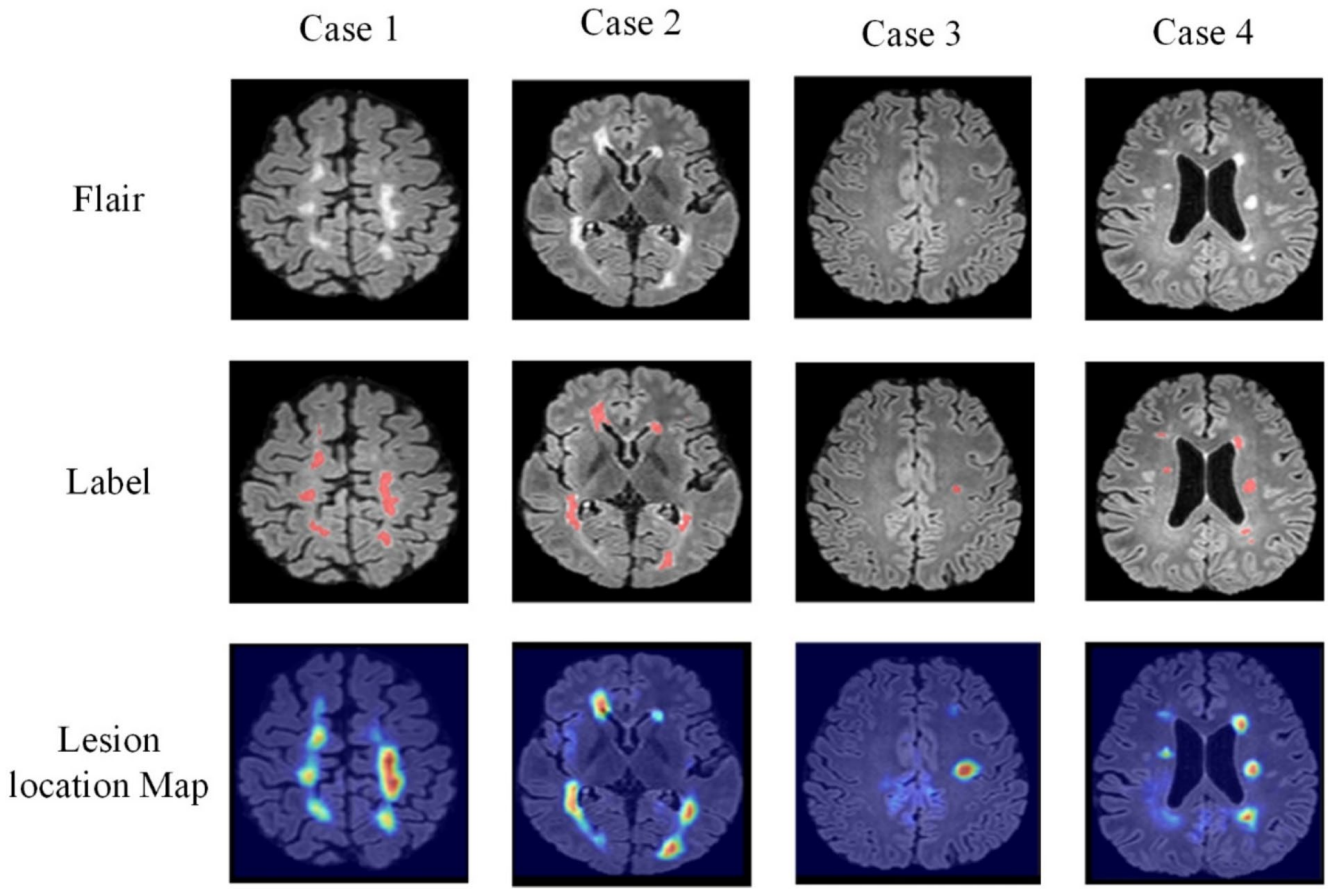
Visual display of lesion localization maps.

In addition, Figure 12 illustrates the comparison between lesion localization maps generated by the joint model and the annotated lesion gold standard for four test samples. The lesion localization maps visualize the model’s focus areas during classification using Grad-CAM (Wagner et al., 2019) technology, where deeper colors indicate higher model attention. Comparing the visualized results with manually annotated segmentation gold standards reveals a substantial alignment between the areas the model emphasizes during classification and the actual lesion locations. Particularly noteworthy is the accurate localization of minute dot-like lesions present in Sample 3, which represent lesions challenging for the segmentation model to distinguish. However, the lesion localization maps manage to accurately pinpoint these lesions. This further validates the reliability of guiding the segmentation model through cross-task losses.

Simultaneously, these lesion localization maps offer interpretability for the joint model in diagnosing MS and NMOSD. They can serve as a basis for deriving diagnostic conclusions for MS and NMOSD in clinical practice.

## 5 Conclusion

This study proposes a joint model for lesion segmentation and disease classification of MS and NMOSD. Leveraging the correlation between lesion segmentation and disease classification tasks, the model facilitates mutual guidance between the two tasks through information sharing and interaction. This approach allows for the effective utilization of the information from limited datasets. Furthermore, comparative experiments confirm the joint model’s ability to significantly enhance the performance of both tasks. Ablation experiments validate the effectiveness of information sharing and interaction mechanisms within the joint model. While the joint model exhibits strong performance in lesion segmentation and disease classification tasks for MS and NMOSD, its generalization capability to other diseases remains limited and somewhat unstable. Therefore, we plan to gather more extensive datasets to enhance the model’s generalizability. Additionally, utilizing multimodal data as input to the model aims to augment its practical applicability.

## Data availability statement

The data analyzed in this study is subject to the following licenses/restrictions: the ISBI dataset used in this study is publicly available: https://smart-stats-tools.org/lesion-challenge-2015. However, the dataset from the First Hospital of Jilin University cannot be made public due to ethical considerations. For access to this dataset, please contact the corresponding author. Requests to access these datasets should be directed to guocj@jlu.edu.cn.

## Author contributions

LH: Supervision, Writing – review & editing. YS: Methodology, Writing – original draft. HY: Supervision, Writing – review & editing. CG: Data curation, Writing – review & editing. YW: Supervision, Writing – review & editing. ZZ: Data curation, Writing – review & editing. YG: Methodology, Writing – original draft.
